# Puerperal Eclampsia: Its Etiology and Treatment1Read before the Medical Society of the State of New York, at Albany, January 26, 1897.

**Published:** 1897-09

**Authors:** William Warren Potter

**Affiliations:** Buffalo, N. Y., Examiner in obstetrics, New York State Medical Examining and Licensing Board; Fellow of the American Association of Obstetricians and Gynecologists


### BUFFALO MEDICAL JOURNAL.

VOL. XXXVII. SEPTEMBER, 1897. No. 2.

### Original Communications.

### PUERPERAL ECLAMPSIA: ITS ETIOLOGY AND TREATMENT.[1]

BY WILLIAM WARREN POTTER, M. D., Buffalo, N. Y.,

Examiner in obstetrics, New York State Medical Examining and Licensing Board; Fellow of the American Association of Obstetricians and Gynecologists.

THE consideration of puerperal eclampsia has been practically a closed book for the past few years, but now, all at once, following the lead of the German school and a few others, a veritable epidemic of society papers and magazine articles has burst upon us. We seem to have arrived at a period that may fittingly be called the renaissance of eclamptic literature, though it is doubtful if great progress is making either in solving problems of causation or treatment of this baneful malady. Nevertheless, it will not answer for this society to keep silent on so important a subject when it seems to be in fashion to discuss it.

Eclampsia ever has been since the birth of the obstetric art one of the most direful calamities that can befall a pregnant woman. There is only one other complication of the lying-in room that approaches it, and that is postpartum hemorrhage. Under present methods, however, the latter has ceased in general to appal the experienced obstetrician, yet the beginner may well regard both hemorrhage and eclampsia as the twin spectres of obstetric practice—the *bêtes noires* of the parturient. While, therefore, nowadays the trained obstetrician expects to deal successfully with postpartum hemorrhage, and may, speaking generally, give a favorable prognosis, not so in eclampsia. Here he is as doubtful of the prognosis as ever, because he can never be certain that the fits may not be renewed, even fatally, until evidences of toxemia have declined and convalescence is well under way. The disappointments and surprises, too, that follow in the trail of eclampsia

1. Read before the Medical Society of the State of New York, at Albany, January 26, 1897.

justify the most guarded prognosis and prompt always a feeling of uncertainty even in the most optimistic minds.

Now, as ever, the pathogenesis of eclampsia is unsettled. It is not a pathologic entity—only a group of symptoms which is named eclampsia, and which varies in intensity all the way from hysterical twitchings to tetanic spasms and coma. Of one fact, however, we are all now certain—namely, that eclampsia is a condition *sui generis*, that it pertains to the puerperal state alone and is found under no other condition. It has no parallel and is not produced any where in the human economy except in the pregnant woman, or immediately after delivery; the convulsions are reflexes excited by cerebro-spinal, or medullary irritation of a toxemic origin; and anemia as well as plethora is conducive to eclampsia.

The early teachings which recognised and described three varieties of eclampsia — hysterical, epileptic and apoplectic,— were erroneous as to pathology and causation as well as misleading in treatment.

In searching for the causes of eclampsia the kidney has always been looked upon with extreme suspicion by a great majority of investigators. It is the duty of the kidneys to eliminate toxins, and if these organs fail in their office unmistakable symptoms are promptly presented in the pregnant woman. These prodromes are manifested in various ways, the leading or most pronounced being headache, puffiness of the eyelids, local and general edema, scanty and albuminous urine. Renal insufficiency, either in quantity or quality, or both, is an almost universal accompaniment of the eclamptic state. Indeed, anurea is often a forerunner of the fits. But the kidney is only a servant,—in the majority of instances, too, healthy in itself,—performing its duties, however, in a faulty manner. It has perhaps been asked to perform excessive labor because of an over-production of toxic material; again by reason of mechanical pressure on its emulgent veins it finds itself embarrassed in its work, or, finally, it may be the seat of parenchymatous change. In any event the kidney plays an important part in the economy of the eclamptic; it is a sentinel stationed at an important gateway which must challenge every toxic element that approaches and eliminate it with promptitude.

In case of over-production of toxins and under-elimination by the kidney, the road is a short one to the eclamptic seizure. The degree of intoxication regulates the severity of the attack, while the nature of the soil in which the toxic elements are sown governs the quality of the seizure. Some women are easily influenced by the eclamptic toxins, while others resist them to the last extremity. In this latter class prodromata are frequently noted and eclampsia is prognosticated, but it rarely comes, or if it does, it is more readily overcome by appropriate measures. I wish it were possible in the present state of our knowledge to fix or differentiate precisely what these mysterious toxins are. If their source, even, were better understood we might do more efficient work in their elimination. It is probable, in my view, that they come in large groups from the intestinal tract, either the result of putrefaction or waste; that they enter the blood in excess of what the kidneys can eliminate, finally causing eclampsia in those women whose systems are easily wrought upon by this group of phenomena.

It is a well-known fact that pregnancy causes a modification of woman's economy often to an extreme degree. It exaggerates her nervous force and diminishes her resisting power; it perverts her mental stamina and lessens her physical energy. The functions of organs whose province is especially to eliminate toxins and so preserve health, are now so disturbed as to prevent the exercise of their offices to their full extent, hence she easily falls a prey to toxemia.

I attach very little importance to the mere presence of albumin in the urine of a pregnant woman, except as a warning that should induce careful watchfulness. How many women with albuminuria escape eclampsia? and, again, how many eclamptics fail to exhibit albuminous urine? Yet it must not be forgotten that a large percentage of albumin means less elimination of toxins, and such a state may become, under favoring conditions, the avant-courier of an eclamptic seizure of maximum intensity.

I shall not take time in this presence to discuss the microbic theory of eclampsia, for it is a proposition that remains to be demonstrated. Moreover, if in the present state of our knowledge we adhere to the belief that toxemia is a causative factor we shall find ourselves on more substantial foundations as to successful treatment.

*Treatment.*—In order to arrive at an intelligent treatment there must be a clear understanding of the conditions we are called upon to treat. Let it be understood, then, that we are dealing with a subtle toxemia not yet understood as to origin or material, nor even as to its *modus operandi*, but still one that arises from ingesta, intestinal putrefaction and fetal metabolism, one or all, and that the organs of elimination are either sluggish or suspended in action. Symptomatically we usually have to contend with severe headache, edema, albuminous and scanty urine, diminished urea excretion ; and, finally, cyanosis, convulsions and coma, or semi-coma. The first group of symptoms pertain to the preëclamptic state and the last to true eclampsia.

Again, the woman may be anemic or plethoric, young or middle-aged, a primipara or multipara and the symptoms grave or moderate in their manifestations. Finally, the eclamptic seizure may be antepartum, intrapartum or postpartum. All these factors serve to modify or control the plan of the treatment to be proposed in a given case.

The treatment of eclampsia, too, should be classified into (*a*) preventive and (*b*) curative. The preventive treatment may be subdivided into medicinal and hygienic, and the curative into medicinal and obstetric.

The preventive treatment of eclampsia affords an interesting field in which the clinician may display his talent and ingenuity in the application of hygienic measures and drugs to avert an impending danger of the gravest import. Given a pregnant woman in the seventh or eighth month with the prodomes of eclampsia, that is to say, who manifests the phenomena incident to the preëclamptic state, and what shall be done ?

Manifestly, the first duty will be to interrogate the kidney as to its sufficiency and integrity. A qualitative and quantitative analysis of the urine hence must be made at the outset. Albuminuria is not a reliable symptom of renal disease or insufficiency, nor is a scanty twenty-four hours' output an unfailing index of kidney failure ; nor yet is a diminution of urea excretion an infallible indication of approaching eclampsia. Exceptionally all of these conditions may coëxist and yet eclampsia not result. These are indications singly and collectively that there is existing toxemia, that there is defective elimination and that something must be done to correct a faulty relationship between nutrition and excretion.

If the prodomes and the physical signs are recognised early, it may be expected with reason that hygiene and medicine will correct the errors that are so rapidly tending toward eclampsia. Air, food and drink must be supplied in ample quantities and of good quality ; so, too, must we insist upon exercise, active or passive—walking, driving, light calisthenics or massage, according to the taste or tolerance of the patient. These are all good agents to employ, but the object sought should be to limit the source of toxins that are being absorbed and to promote their elimination.

One of the surest ways in which to control the supply of toxins appears to be, from abundant testimony, to place the woman upon an exclusively milk diet. This will serve at once not only to diminish the supply of toxins, but to increase the fluids of the body, flush the kidneys and favor the elimination of toxic material. Water, too, should be freely given in definite quantities and at regular intervals. I speak from a considerable experience when I say that distilled water is one of the best diuretics that can be administered to a woman in the eclamptic state. Two quarts a day is not too much and it may be given still or charged, according to the taste or desire of the patient.

The bowels of the preëclamptic, too, demand supervision. Constipation must not only be prevented, but intestinal toxins must be unloaded, and the intestinal tract kept free. These are common-place observations perhaps, but they are essentials that cannot be omitted in a consecutive clinical picture of the management of a woman with eclamptic prodromes. Drugs are not specified in kind, but each physician will invoke the aid of an intelligent pharmacology. It should be remembered, however, that forcing the kidneys without supplying copious fluids is to be reprehended. Potassium salts that have been so frequently employed, and as I believe to the detriment of eclamptic patients, should be avoided. They favor the production of intestinal toxins, and beside tend to diminish red blood corpuscles—an element that must be conserved.

There are two other remedies of which I wish to speak just here, for they belong to the therapeutics of the condition we are discussing, that is, the preëclamptic state. These are blood-letting and glonoin. If there is a full artery at the wrist with a tendency to cyanosis in the preëclamptic, venesection may be resorted to with benefit. One good full bleeding is permissible, but it should be used with caution in repetition. I fear its employment during an eclamptic seizure, but here it is admissible, and in selected cases it will often prove beneficial. If there is high arterial tension—vasomotor spasm—glonoin in full doses is a valuable remedy. It combats this condition without depleting the patient, and, moreover, helps to set the kidney to work. Let us now dismiss the preëclamptic state and consider the treatment of true eclampsia.

Suppose a physician is summoned as a consultant in a case where convulsions have already set in, what is to be done? Here let us recall the three forms of eclampsia, (*a*) antepartum, (*b*) intrapartum, (*c*) postpartum. The treatment will be, first, medicinal and, second, obstetric. The first indication of treatment in antepartum eclampsia is to control the convulsions. An attempt wisely enough may be made to do this through the administration of chloroform by inhalation as well as the administration of chloral by the rectum. An eclamptic woman swallows with difficulty or not at all, as she rarely rouses to full consciousness between the fits. Hence, it is better to administer chloral by the rectum. For my part I would administer chloroform rather tentatively in these cases. If the convulsions were not promptly controlled or diminished in frequency or severity by its vigorous and skilful administration, I should institute measures at once to empty the uterus of its contents; and this brings us face to face with the most interesting question in the obstetric management of puerperal eclampsia.

A considerable experience has convinced me that a prompt evacuation of the uterus constitutes the most important method of dealing with eclampsia. While the womb remains gravid we are hampered in our therapeutics. Two lives are at stake and in our anxiety to preserve both we may save neither. By addressing ourselves assiduously to a speedy delivery of the fetus we contribute in the largest manner to the conservation of both lives. With the fetus once delivered there is a freer opportunity to deal with the woman in the most masterful or even heroic manner. Fortunately eclampsia rarely occurs until the fetus has reached the period of viability; hence its speedy delivery becomes its greatest safeguard; for every hour increases its liability to death from maternal toxemia, as well as diminishes its chances of survival after premature delivery.

How many times has the fetus been destroyed by prolonged chloroform anesthesia, full morphinism, or extreme chloralisation? One or all of these means, to be sure, may be used with comparative safety to the mother if the fetus is out of the way. How often, too, has the fetus succumbed to prolonged intoxication from the mother's blood?

If I am called to an eclamptic woman who is within a month of term I lay down as a cardinal principle for my own guidance that it is my duty to proceed with all diligence to effect delivery. Why should not this be done? Will any one tell us why it is not good obstetric practice to proceed to aid nature in the accomplishment of her own desires? I regard the appearance of eclampsia as an expression of the economy that it has carried an offending fetus as long as it can be tolerated. The toxemia incident to its presence can no longer be endured without calamitous results, and eclampsia is but an expression of that fact—a danger signal. If we should appeal to statistics they would tell us that the mortality in antepartum eclampsia is seven times greater than in postpartum eclampsia. Surely this speaks in no uncertain fashion commendatory of speedy delivery. But we are told that convulsions do not always cease after delivery. True, but is that a valid argument against the induction of labor? Postpartum hemorrhage does not always cease after the delivery of the placenta and secundines, but who will affirm that it is not proper in such a case to begin the treatment by clearing out the uterus? If convulsions continue after delivery we are now in a better position to push medicinal treatment, if need be, to its extreme limit than before. Purgatives, morphine, chloral, chloroform, diuretics and diaphoretics may all of them be employed with greater expectations and less danger than before delivery.

I have induced labor in a number of instances near the end of the eighth month and always with most satisfactory result. In several of these cases, girls and boys now in the heyday of youth are living to gladden the hearts of mothers also rescued from a desperate strait. In no one instance have I ever regretted the induction of labor, while, on the other hand, I recall a few times in which I blame myself for not invoking its aid. Occasionally, too, I have induced labor where there has been exaggerated preëclamptic conditions without convulsions—edema, albuminuria, semi-coma and impending danger. These also have afforded gratifying results in living children and cured mothers.

Having determined to evacuate the uterus, how shall it be done? There are several methods recommended, but now one and then another seems applicable to a given case. For the most part in antepartum eclampsia, dilatation, first practised with steel dilators, if need be, then with manual stretching of the os and cervix, will accomplish the work to the best advantage. Exceptionally, however, extreme measures may become necessary, but only rarely can the deep incisions of Dührssen be required. So, too, should Cesarean section be reserved for extreme complications like deformed pelvis, or to be done in the interest of the fetus when the mother's condition seems to be hopeless. In antepartum eclampsia, after obtaining complete dilatation, it is a good plan to terminate labor at once with the forceps under profound anesthesia.

In postpartum eclampsia the treatment must of necessity be medicinal, just as it must also be in the antepartum variety when convulsions continue after delivery. I have purposely avoided including veratrum viride in the group of medicinal agents which I would employ, because I deem it dangerous, uncertain and deceptive in its action. It is but a symptomatic remedy at best, and it has always seemed to me that its employment was analogous to that of antipyretics in typhoid fever or pneumonia. These dangerous drugs seek to reduce the temperature at the expense of cardiac force without in any way exercising any influence over the real course of the disease. So, too, with veratrum in eclampsia. It reduces arterial tension and cardiac pressure without exercising special influence over the progress of the malady. Moreover, it may easily be given to a dangerous degree even by careful hands. I am very much afraid that many cases of eclampsia have succumbed to the indiscreet employment of veratrum, chloroform, chloral and other powerful cardiac depressants ; or, in other words, that these agents, though producing physiological action and apparently controlling the convulsions, yet do so at the expense of the tone and force of the heart-muscle.

Blood-letting for similar reasons should be discreetly employed and, in my opinion, limited to a few cases of the antepartum variety with plethoric habits and manifesting cyanosis. In the medicinal treatment principles are only discussed and not details. These latter will easily be supplied by experienced and well-trained physicians.

There remains one variety of eclampsia to be mentioned—namely, that of pregnancy as distinguished from eclampsia appearing just before, during or after delivery. This form though representing, statistically speaking, the most dangerous variety, nevertheless is one in which there is more time for deliberate action. It is here, too, that medicinal treatment offers better promise, hence the question of evacuating the uterus may be deferred until the other manifests failure or inadequacy.

Premature delivery may be induced in these cases by passing an aseptic bougie well up the fundus uteri externally to the membranes. Its extremity should be coiled within the vagina and retained by antiseptic packing. In a few hours labor will set in and can then be terminated rapidly if the occasion should so demand. Eclampsia occurs proportionately with greater frequency as the woman advances toward term, and accouchement forcé is more easily put into practice in the antepartum variety.

Finally, to promote the elimination of toxic material the value of diuresis, catharsis and diaphoresis should not be forgotten; neither should the means of the hot-air bath and the hot pack be overlooked. Let me say in conclusion that my aim in this paper has been to present practical experience rather than theoretical views. Hence I have avoided quotations from text-books or magazines, eliminating statistics and omitting tedious reports of cases. Some of my own cases have been published and others have not. Perhaps I may some day group them and give deductions therefrom in a supplementary paper. In this connection I desire to call attention to the fact that two papers have lately appeared on eclampsia that deserve careful study. One of these was presented by Charpentier, of Paris, at the Geneva Congress of Obstetrics and Gynecology in September, 1896. This paper antagonises very forcibly the practice of speedily emptying the uterus as a primary resource. The other paper by Clifton Edgar, of New York, published in the *Medical Record*, December 26, 1896, takes the opposite view, advocating the evacuation of the uterus as a principal means of cure. Both of these papers are masterful presentations of divergent views on this point.

I desire to array myself on the side of Edgar, with whom I agree upon all essentials in his dissertation. His views correspond more nearly with my own experience. His paper is a decided contribution to the literature of eclampsia.

284 Franklin Street.

